# Activation of SK/K_Ca_ Channel Attenuates Spinal Cord Ischemia-Reperfusion Injury via Anti-oxidative Activity and Inhibition of Mitochondrial Dysfunction in Rabbits

**DOI:** 10.3389/fphar.2019.00325

**Published:** 2019-04-02

**Authors:** Jie Zhu, Li-Kun Yang, Wei-Liang Chen, Wei Lin, Yu-Hai Wang, Tao Chen

**Affiliations:** Department of Neurosurgery, The 101th Hospital of PLA, School of Medicine, Anhui Medical University, Wuxi, China

**Keywords:** SK/K_Ca_ channels, NS309, spinal cord ischemia and reperfusion, mitochondrial dysfunction, mitochondrial dynamic

## Abstract

Spinal cord ischemia-reperfusion injury (SCI/R) is a rare but devastating disorder with a poor prognosis. Small conductance calcium-activated K^+^ (SK/K_Ca_) channels are a family of voltage-independent potassium channels that are shown to participate in the pathological process of several neurological disorders. The aim of this study was to investigate the role of SK/K_Ca_ channels in experimental SCI/R in rabbits. The expression of SK/K_Ca_1 protein significantly decreased in both cytoplasm and mitochondria in spinal cord tissues after SCI/R. Treatment with 2 mg/kg NS309, a pharmacological activator for SK/K_Ca_ channel, attenuated SCI/R-induced neuronal loss, spinal cord edema and neurological dysfunction. These effects were still observed when the administration was delayed by 6 h after SCI/R initiation. NS309 decreased the levels of oxidative products and promoted activities of antioxidant enzymes in both serum and spinal cord tissues. The results of ELISA assay showed that NS309 markedly decreased levels of pro-inflammatory cytokines while increased anti-inflammatory cytokines levels after SCI/R. In addition, treatment with NS309 was shown to preserve mitochondrial respiratory complexes activities and enhance mitochondrial biogenesis. The results of western blot analysis showed that NS309 differentially regulated the expression of mitochondrial dynamic proteins. In summary, our results demonstrated that the SK/K_Ca_ channel activator NS309 protects against SCI/R via anti-oxidative activity and inhibition of mitochondrial dysfunction, indicating a therapeutic potential of NS309 for SCI/R.

## Introduction

Spinal cord ischemia-reperfusion injury (SCI/R) is a rare but devastating disorder induced by a period of deterioration of the spinal cord blood supply. SCI/R is associated with several pathophysiological states, while thoracoabdominal surgery and atherosclerotic disease are the most frequent causes (New and McFarlane, 2012). It has been shown that approximately 32% of patients with thoracic or thoracoabdominal aortic repair surgery suffered from SCI/R, and the incidence of paraplegia was up to 5% (LeMaire et al., 2012; Panthee and Ono, 2015). Many neuroprotective drugs, such as mannitol, corticosteroids and naloxone, have been demonstrated to be beneficial to function recovery in experimental SCI/R models, but none of them was convincingly confirmed in clinical trials (Griepp and Griepp, 2007).

Small conductance calcium-activated K^+^ (SK/K_Ca_) channels are a family of voltage-independent potassium channels that are activated solely by intracellular Ca^2+^. SK/K_Ca_ channels are extensively expressed throughout the central nervous system, including the brain and spinal cord tissues (Kuiper et al., 2012). They are encoded by the *KCNN* gene family, and three different subtypes of SK/K_Ca_ channels, including SK/K_Ca_1, SK/K_Ca_2, and SK/K_Ca_3, have been cloned in mammals (Stocker, 2004). SK/K_Ca_ channels are activated by increases in intracellular Ca^2+^, and they not only contribute to the after-hyperpolarization that follows action potentials, but also play key roles in regulating dendritic excitability, synaptic transmission and synaptic plasticity (Faber and Sah, 2007). Three decades of research has shown that SK/K_Ca_ channels are involved in multiple neurological diseases caused by neuronal excitotoxicity and dysfunction of Ca^2+^ homeostasis (Kuiper et al., 2012). By using pharmacological inhibitors and activators, Dolga and colleagues found that SK/K_Ca_ channels participated in the pathological process of stroke, Parkinson’s disease (PD), Alzheimer’s disease (AD) and schizophrenia (Anderson et al., 2006; Dolga et al., 2011, 2012; Dolga and Culmsee, 2012). A previous study showed that activation of the SK/K_Ca_ channel protected against glutamate-induced oxytosis through inhibiting mitochondrial dysfunction (Dolga et al., 2013). More importantly, activation of the SK/K_Ca_ channel in the spinal cord was shown to reduce the NMDA receptor antagonist dose needed to produce anti-nociception in an inflammatory pain model (Hipolito et al., 2015). In the present study, we investigated the role of SK/K_Ca_ channels in a SCI/R model in rabbits. Previous studies have demonstrated that SK/K_Ca_ channels were also located in mitochondrial-enriched fraction, and this type of subcellular expression of SK/K_Ca_ channels was associated with normal mitochondrial function in dopaminergic neurons (Dolga et al., 2014). Thus, we also determined the underlying mechanism with focus on oxidative stress and mitochondrial function.

## Materials and Methods

### Animals and Agents

Adult New Zealand white rabbits (2.5–3.0 kg) were obtained from the Laboratory Animal Center of Anhui Medical University. All rabbits were kept in standard laboratory conditions with free access to rabbit chow and tap water. All experimental procedures were performed in compliance with the NIH guidelines for the use of experimental animals and approved by the Ethics Review Committee of Anhui Medical University. The mitochondrial isolation kit, malondialdehyde (MDA) assay kit, 8-iso-prostaglandin F2α (8-iso-PGF2α) assay kit, superoxide dismutase (SOD) assay kit and catalase (CAT) assay kit was purchased from Jiancheng Biotech Company (Nanjing, Jiangsu, China). The anti-SK/K_Ca_1 (ab66624), anti-COX IV (ab66739), and anti-Tubulin (ab56676) antibodies were obtained from abcam (CA, United States). The anti-Opa-1 (sc-30573), anti-Mfn-1 (sc-166644), anti-Drp-1 (sc-101270), anti-Fis-1 (sc-376447), and anti-β-actin (sc-47778) antibodies were purchased from Santa Cruz Biotechnology (Santa Cruz, CA, United States).

### SCI/R Model

SCI/R was established by infrarenal aortic occlusion as previously described (Zhou et al., 2013). The animals were anesthetized by intramuscular administration of 25 mg/kg ketamine and 0.10 mg/kg atropine. The right femoral artery was catheterized for measuring distal blood pressure and ear artery was used to collect blood sample. A 5-cm-long midline incision was made to expose abdominal aorta, and aortic clamping was performed using artery clips just below renal artery after administration of 200 units of heparin. After 20 min occlusion, the clips were removed to restore the blood flow. Then, the incision was sutured layer-by-layer, and the rabbits were allowed to recover. During the operation process, the body temperature was maintained close to 38.2°C using a heating lamp.

### Experimental Design

#### Experimental 1

To investigate the effect of SCI/R on the expression of SK/K_Ca_1 and mitochondrial dynamic proteins, forty animals were randomly divided into sham (*n* = 8) and SCI/R (*n* = 32) group. The animals in sham group were given operation without artery occlusion. The animals in SCI/R group were treated with SCI/R and spinal cord tissues were obtained at 3, 6, 12, and 24 h.

#### Experimental 2

To investigate the protective effect of NS309 against SCI/R, thirty-two animals were randomly divided into vehicle (*n* = 16) and NS309 (*n* = 16) group. The animals in NS309 group were treated with 2 mg/kg NS309 by intraperitoneal injection at the time of spinal cord ischemia initiation, and the animals in vehicle group were treated with saline (0.9%) with 1% DMSO. Eight animals in each group were used to measure neurological function and H&E staining, and the other 8 animals were used to measure spinal cord edema.

#### Experimental 3

To investigate the therapeutic time window of NS309, sixty-four animals were randomly divided into vehicle (*n* = 16), NS309 at 3 h (*n* = 16), NS39 at 6 h (*n* = 16) and NS309 at 9 h (*n* = 16) group. The animals in vehicle group were treated with saline (0.9%) with 1% DMSO, and the animals in NS309 groups were treated with 2 mg/kg NS309 by intraperitoneal injection at 3, 6, or 9 h after spinal cord ischemia initiation, respectively. Eight animals in each group were used to measure neurological function and H&E staining, and the other 8 animals were used to measure spinal cord edema.

#### Experimental 4

To investigate the effect of NS309 on oxidative stress, and mitochondrial dysfunction, sixteen animals were randomly divided into vehicle (*n* = 8) and NS309 (*n* = 8) group. The animals in NS309 group were treated with 2 mg/kg NS309 by intraperitoneal injection at the time of spinal cord ischemia initiation, and the animals in vehicle group were treated with saline (0.9%) with 1% DMSO. The blood samples were collected at 0, 3, 6, 12, 24, and 48 h after spinal cord ischemia initiation, and the spinal cord tissues were collected at 72 h.

### Mitochondrial Isolation

After treatments, the spinal cord tissues were removed and homogenized, and mitochondria was isolated using the mitochondrial isolation kit following the manufacturer’s suggested protocol. Mitochondrial pellets were resuspended, and the protein content was quantified using a BCA method. The mitochondrial and cytosolic fractions were used to detect SK/K_Ca_1 expression, and the total protein was used to detect the expression of mitochondrial dynamic proteins.

### Hematoxylin and Eosin (H&E) Staining

After various treatments, the spinal cord tissues were removed, and the paraffin embedded blocks were made. The 5 μm sections from each group was subjected to standard H&E staining and evaluated microscopically. The number of normal motor neurons was counted by an investigator blinded to the grouping.

### Spinal Cord Edema Measurement

Spinal cord edema was evaluated by spinal cord water content using the wet-dry method at 72 h after SCI/R. Briefly, after the animals were sacrificed, the spinal cord tissues (L4-6 segments) were quickly removed. Tissue samples were weighed immediately to get wet weight. After drying in an oven for 48 h at 100°C, the tissues were re-weighed to get the dry weight. Spinal cord water content was then calculated using the following formula: % H_2_O=(1-dry weight/wet weight) × 100%.

### Neurological Function Assay

The hind-limb motor function was determined by the modified Tarlov criteria: no voluntary hind-limb function as score 0; only perceptible joint movement as score 1; active movement but unable to stand as score 2; able to stand but unable to walk as score 3; and complete normal hind-limb motor function as score 4.

### Sample Collection

At 0, 3, 6, 12, 24, and 48 after SCI/R, 2 mL of blood was collected from auricular vein, and the serum was separated by centrifugation at 3,000 × *g* for 15 min at 4°C. At 72 h after reperfusion, the animals were sacrificed, the spinal cord tissues (L4-6 segments) were removed. The spinal cord samples were homogenized in chilled PBS, and then centrifuged at 10,000 × *g* at 4°C for 10 min. The protein concentration was quantified using a BCA method.

### Quantification of Oxidative Products and Antioxidant Enzymes

The levels MDA and 8-iso-PGF2α and the enzymatic activities of SOD and CAT in the serum and spinal cord samples were measured by commercial kits following the manufacturer’s suggested protocol.

### Quantification of Inflammatory Cytokines

To detect the levels of inflammatory cytokines, animals were sacrificed at 12, 24, or 48 h after SCI/R, and the spinal cord homogenates were obtained. The concentrations of tumor necrosis factor-α (TNF-α), IL-1β, IL-10 and transforming growth factor-β1 (TGF-β1) were measured using specific ELISA kits according to the manufacturers’ instructions (Boster Biological Technology, Wuhan, China).

### Determination of Mitochondrial Complexes Activities

After treatment with NS309 or vehicle, spinal cord mitochondria were isolated from each group and the enzymatic activities of mitochondrial complexes were assayed using the following methods: NADH dehydrogenase for complex I, succinate dehydrogenase for complex II, ubiquinol cytochrome c oxidase for complex III, and cytochrome c oxidase for complex IV as previously described (Ye et al., 2011).

### Real-Time RT-PCR

Total RNA was extracted from the spinal cord tissues using the Trizol reagent (Invitrogen, Carlsbad, CA, United States). Reverse transcription was performed according to the manufacturer’s instructions of the real-time PCR kit (BioTNT Co., Ltd., Shanghai, China). The primers sequences used are shown in Table 1. The mRNA levels were normalized using GAPDH as an internal control.

**Table 1:** Primers sequences used in real-time PCR.

Gene	Forward sequences	Reverse sequences
D-loop	5′-AGGCATCTGGTTCTTACTTC-3′	5′ -TGACGGCTATGTTGAGGA-3′
ATP8	5′-CTTCCCAAACCTTTCCTG-3′	5′ -GGTAATGAAAGAGGCAAATAGA-3′
PGC-1	5′-GTGCAGCCAAGACTCTGTATGG-3′	5′ -GTCCAGGTCATTCACATCAAGTTC-3′
NRF-1	5′-GAGTGACCCAAACCGAACA-3′	5′ -GGAGTTGAGTATGTCCGAGT-3′
TFAM	5′-GGTGTATGAAGCGGATTT-3′	5′ -CTTTCTTCTTTAGGCGTTT-3′
GAPDH	5′-ATGTATCCGTTGTGGATCTGAC-3′	5′ -CCTGCTTCACCACCTTCTTG-3′

### Western Blot Analysis

Total proteins from spinal cord tissues were extracted and the protein concentration was determined using a BCA assay kit (Jiancheng Biotech Company, Jiangsu, China). Equivalent proteins (60 μg per sample) were separated using 10 or 12% sodium dodecyl sulfate (SDS)-PAGE, and then electro-transferred onto polyvinylidene fluoride (PVDF) membranes. The membranes were incubated with the following primary antibodies: SK/K_Ca_1 (1:500), COX IV (1:800), Tubulin (1:1000), Opa-1 (1:500), Mfn-1 (1:800), Drp-1 (1:800), Fis-1 (1:500), and β-actin (1:2000). After incubation with secondary antibodies for 1 h, the bands were visualized by using chemiluminescent detection system. Image J (Scion Corporation) was used to quantify the optical density of each band. The expression of each protein was calculated from the optical density of each band normalized against the optical density of β-actin, tubulin or COX IV, and expressed as the fold of control levels.

### Statistical Analysis

Each experiment was repeated at least three times. Statistical analysis was performed using SPSS. Statistical evaluation of the data was performed by the Student’s *t*-test between two groups. A value of *p* < 0.05 referred to the statistical difference.

## Results

### SCI/R Decreases the Expression of SK/K_Ca_1 Subtype

The changes of arterial pH, PaO_2_, PaCO_2_, and blood glucose levels in vehicle or 2 mg/kg NS309 treated rabbits were measured at 10 min before ischemia, 10 min after ischemia initiation and 10 min after reperfusion, respectively. As shown in Table 2, no significant differences in blood pH, PaO_2_, PaCO_2_, or glucose were observed among all groups. The results of distal blood pressure (DBP) showed that an approximate 90% reduction in DBP was observed during the time when artery was clamped. The value of DBP was recovered to 90–95% of the baseline within 10 min after reperfusion.

**Table 2:** Physiological parameters.

	Rectal temperature (°C)	pH	PaO_2_ (mm Hg)	PaCO_2_ (mm Hg)	Glucose (mg/dl)	DBP (mm Hg)
*10 min before ischemia*						
Vehicle	38.3 ± 0.2	7.41 ± 0.13	124.2 ± 11.4	34.4 ± 3.8	157 ± 17	106 ± 5.7
NS309 2 mg/kg	38.1 ± 0.2	7.39 ± 0.09	129.5 ± 13.9	31.1 ± 4.6	168 ± 19	104 ± 7.1
*10 min after ischemia*						
Vehicle	38.3 ± 0.3	7.35 ± 0.19	133.4 ± 15.3	34.3 ± 2.5	154 ± 18	13 ± 2.9^∗^
NS309 2 mg/kg	38.2 ± 0.2	7.38 ± 0.17	127.9 ± 15.2	32.7 ± 4.3	164 ± 23	17 ± 4.3^∗^
*10 min after reperfusion*						
Vehicle	38.0 ± 0.1	7.37 ± 0.10	125.9 ± 11.4	36.8 ± 3.3	153 ± 19	100 ± 10.4
NS309 2 mg/kg	38.4 ± 0.4	7.41 ± 0.18	131.6 ± 12.3	38.3 ± 3.1	160 ± 22	98 ± 9.3

*Data are presented as mean ± SEM. ^∗^*p* < 0.05 vs. 10 min before ischemia.*

**FIGURE 1:**
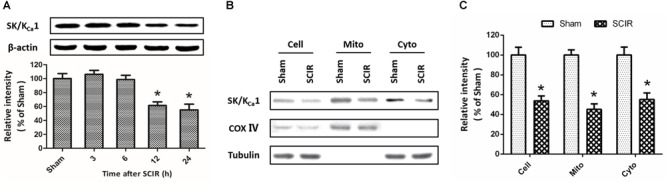
SCI/R decreases the expression of SK/K_Ca_1 subtypes. The animals were exposed to spinal I/R, and the expression of SK/K_Ca_1 protein in spinal cord tissues was detected by western blot analysis at 3, 6, 12, or 24 h after reperfusion **(A)**. Immunoblot analysis of whole-cell extract (depicted as “Cell”), cytosol supernatant (depicted as “Cyto”) or crude mitochondrial pellets (depicted as “Mito”) were also performed **(B)**, and calculated **(C)** to determine subcellular expression of SK/K_Ca_1 protein. Data are shown as mean ± SEM (*n* = 8). ^∗^*p* < 0.05 vs. Sham.

To investigate the effect of SCI/R on SK/K_Ca_ channels expression, western blot analysis was used to detect the changes of SK/K_Ca_1 subtype at 3, 6, 12, or 24 h after reperfusion (Figure 1A). The results showed that the expression of SK/K_Ca_1 protein significantly decreased at 12 and 24 h after reperfusion. To determine the subcellular localization of SK/K_Ca_1 after SCI/R, the expression of SK/K_Ca_1 protein was assayed after mitochondria isolation (Figure 1B). The results showed an enrichment of SK/K_Ca_1 from whole cell extracts to cytoplasm, as well as in mitochondrial fractions, and the decreased expression of SK/K_Ca_1 protein was also observed in isolated mitochondria after SCI/R (Figure 1C).

### The SK/K_Ca_ Channel Activator NS309 Protects Against SCI/R

**FIGURE 2:**
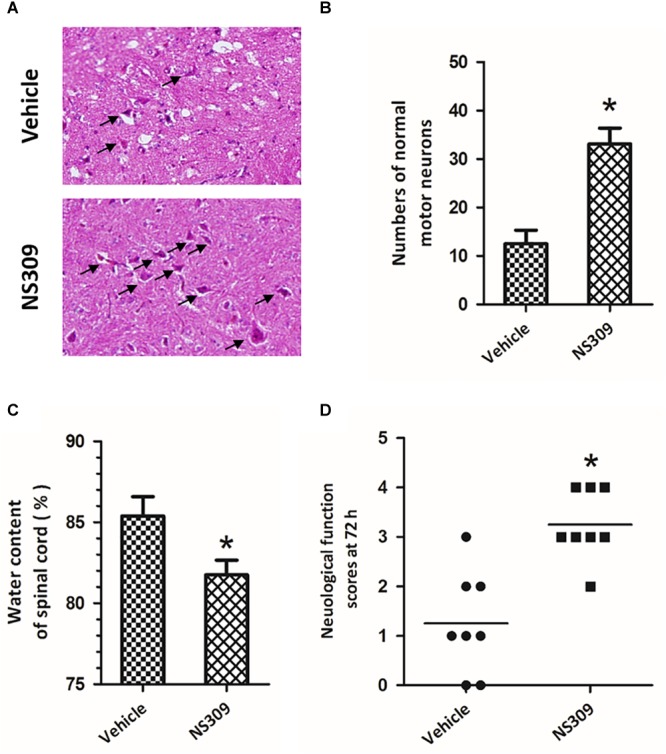
The SK/K_Ca_ channel activator NS309 protects against SCI/R. The animals were treated with 2 mg/kg NS309 or vehicle at the beginning of SCI/R operation. Hematoxylin and eosin staining was performed in the ventral horn of L4 spinal cord segment at 72 h after reperfusion **(A)**, and the number of normal motor neurons was countered **(B)**. The spinal cord edema was assayed by measuring spinal cord water content at 72 h **(C)**. Neurological function scores were assessed at 72 h after reperfusion by an independent observer **(D)**. Arrowheads indicate normal neurons. The data were represented as means ± SEM (*n* = 8). ^∗^*p* < 0.05 vs. Vehicle.

To investigate the effect of SK/K_Ca_ channel activation on SCI/R, rabbits were treated with 2 mg/kg NS309, a pharmacological activator for SK/K_Ca_ channel, at the time of spinal cord ischemia initiation. The representative micrographs of H&E staining in the ventral horn of L4 spinal cord segment at 72 h after reperfusion are shown in Figure 2A. The number of normal motor neurons in NS309 group was higher than that in vehicle group (Figure 2B). We also determined spinal cord edema by measuring water content of spinal cord tissues at 72 h after injury, and a marked decrease in spinal cord water content was observed in NS309 treated animals as compared with vehicle group (Figure 2C). Next, we measured hind-limb motor function scores at 72 h after injury. As shown in Figure 2D, treatment with NS309 significantly improved neurological outcome of SCI/R challenged rabbits.

**FIGURE 3:**
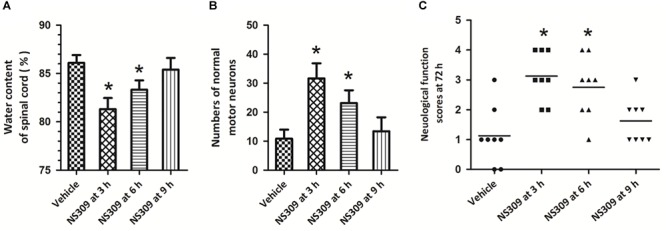
Time window of NS309-induced neuroprotection. The animals were exposed to SCI/R and treated with 2 mg/kg NS309 at different time points (3, 6, or 9 h after SCI/R beginning). The spinal cord water content **(A)**, number of normal motor neurons **(B)** and neurological function scores **(C)** were measured 72 h later. The data were represented as means ± SEM (*n* = 8). ^∗^*p* < 0.05 vs. Vehicle.

To determine the therapeutic time window of NS309-induced protective effects, the post-injury administration pattern was used, and the rabbits were treated with 2 mg/kg NS309 at different time points (3, 6, or 9 h after ischemia initiation). A significant reduction in spinal cord water content (Figure 3A) and increased numbers of normal motor neurons (Figure 3B) were observed when NS309 was administrated 3 or 6 h after ischemia initiation but not when the administration was delayed by 9 h. In consistent with these results, a significant improvement of neurologic outcome was still detected when NS309 treatment was started 3 or 6 h, but not 9 h after ischemia initiation (Figure 3C).

### NS309 Reduces Oxidative Stress and Regulates Inflammation After SCI/R

**FIGURE 4:**
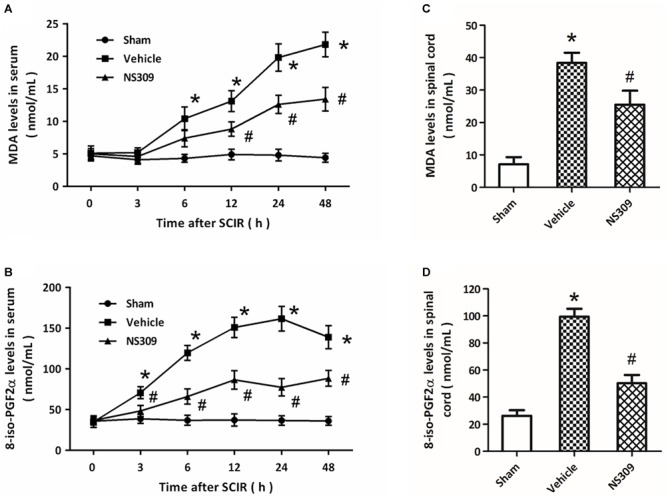
NS309 reduces the expression of oxidative products after SCI/R. The animals were treated with 2 mg/kg NS309 or vehicle at the beginning of SCI/R. The expression levels of MDA **(A)** and 8-iso-PGF2α **(B)** in serum were measured at different time points (0, 3, 6, 12, 24, and 48 h). The expression levels of MDA **(C)** and 8-iso-PGF2α **(D)** in spinal cord tissues were detected at 72 h after reperfusion. The data were represented as means ± SEM (*n* = 8). ^∗^*p* < 0.05 vs. Sham. ^#^*p* < 0.05 vs. Vehicle.

**FIGURE 5:**
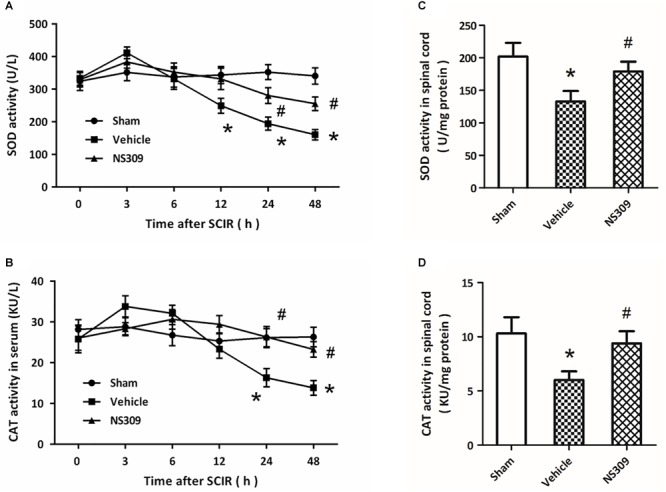
NS309 preserves the activity of antioxidant enzymes after SCI/R. The animals were treated with 2 mg/kg NS309 or vehicle at the beginning of SCI/R. The enzymatic activities of SOD **(A)** and CAT **(B)** in serum were measured at different time points (0, 3, 6, 12, 24, and 48 h). The enzymatic activities of SOD **(C)** and CAT **(D)** in spinal cord tissues were detected at 72 h after reperfusion. The data were represented as means ± SEM (*n* = 8). ^∗^*p* < 0.05 vs. Sham. ^#^*p* < 0.05 vs. Vehicle.

The levels of MDA and 8-iso-PGF2α, two classic oxidative products, in serum and spinal cord tissues were measured after SCI/R. The results showed that the levels of MDA (Figure 4A) and 8-iso-PGF2α (Figure 4B) in serum significantly increased after SCI/R in a time-dependent manner, but these increases were attenuated by NS309 treatment. The levels of MDA (Figure 4C) and 8-iso-PGF2α (Figure 4D) in spinal cord tissues from NS309-treated animals was lower than that in vehicle group. In addition, we also detected the activities of SOD and CAT, two endogenous antioxidant enzymes. The results showed that the activities of SOD (Figure 5A) and CAT (Figure 5B) in serum from NS309-treated animals was higher than that in vehicle group from 12 to 48 h after SCI/R. As shown in Figure 5C,D, NS309 also markedly increased SOD and CAT activities in spinal cord tissues.

**FIGURE 6:**
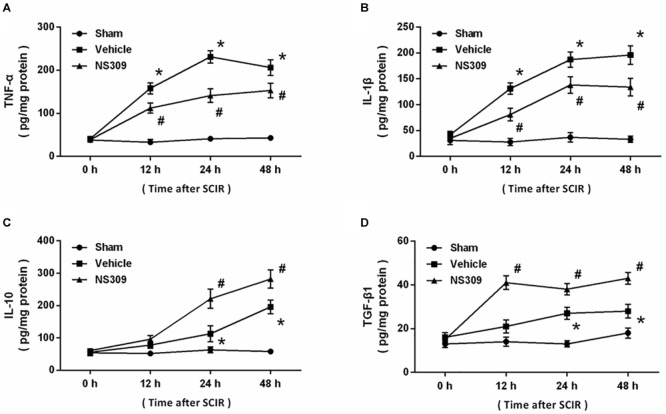
NS309 regulates inflammatory cytokines after SCI/R. The animals were treated with 2 mg/kg NS309 or vehicle at the beginning of SCI/R. The levels of TNF-α **(A)**, IL-1β **(B)**, IL-10 **(C)**, and TGF-β1 **(D)** in spinal cord tissues were determined at different time points (0, 12, 24, and 48 h). The data were represented as means ± SEM (*n* = 8). ^∗^*p* < 0.05 vs. Sham. ^#^*p* < 0.05 vs. Vehicle.

To investigate the effect of NS309 on inflammatory responses after SCI/R, the levels of inflammatory cytokines were determined in spinal cord tissues at different time points. The results showed that SCI/R significantly increased the levels of TNF-α (Figure 6A) and IL-1β (Figure 6B), two pro-inflammatory cytokines. However, application of NS309 significantly reduced the levels of these two cytokines in spinal cord tissues at 12, 24 and 48 h. Furthermore, we also measured the levels of IL-10 and TGF-β1, two anti-inflammatory cytokines. We found that NS309 markedly increased the levels of IL-10 at 24 and 48 h, but not at 12 h after SCI/R (Figure 6C). The TGF-β1 levels in NS309-treated group were higher than that in vehicle group at all time points measured (Figure 6D).

### NS309 Inhibits Mitochondrial Dysfunction After SCI/R

**FIGURE 7:**
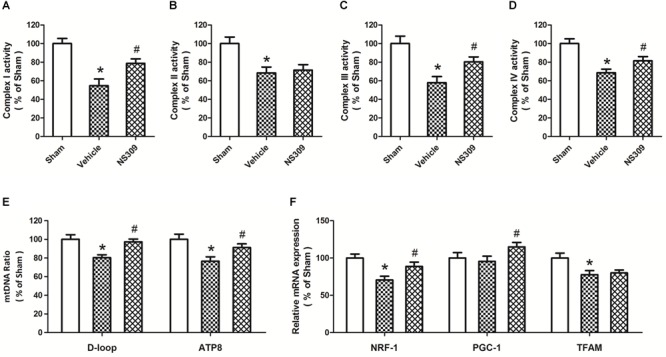
NS309 preserves mitochondrial complex activity and mitochondrial biogenesis after SCI/R. The animals were treated with 2 mg/kg NS309 or vehicle at the beginning of SCI/R. The activities of mitochondrial complex I **(A)**, II **(B)**, III **(C)**, and IV **(D)** were observed in spinal cord, respectively. Real-time RT-PCR was used to detect mtNDA ratio **(E)** and the expression of mitochondrial biogenesis factors **(F)**. The data were represented as means ± SEM (*n* = 8). ^∗^*p* < 0.05 vs. Sham. ^#^*p* < 0.05 vs. Vehicle.

Mitochondrial function plays a key role in regulating oxidative stress under ischemia and reperfusion injury. Thus, we detected the activities of mitochondrial complexes in spinal cord tissues. The results showed that SCI/R significantly decreased the activities of all four mitochondrial complexes, and the activities of complex I (Figure 7A), complex III (Figure 7C) and complex IV (Figure 7D), but not complex II Figure 7B), were preserved by NS309 treatment. In addition, we investigated the effect of NS309 on mitochondrial biogenesis. NS309 increased mtDNA levels (Figure 7E) and elevated the levels of NRF-1 and PGC-1 mRNA after SCI/R (Figure 7F).

**FIGURE 8:**
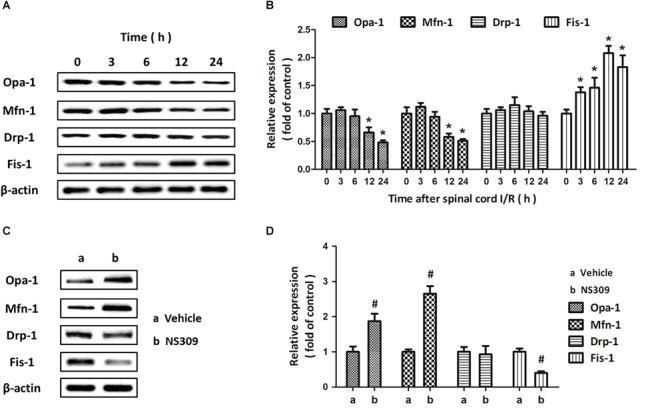
NS309 differently regulates the expression of mitochondrial dynamic proteins. The animals were treated with 2 mg/kg NS309, and the expression of Opa-1, Mfn-1, Drp-1, and Fis-1 at different time points (0, 3, 6, 12, and 24 h) was detected by western blot analysis **(A,B)**. The animals were treated with 2 mg/kg NS309 or vehicle at the beginning of SCI/R. The expression of Opa-1, Mfn-1, Drp-1, and Fis-1 was detected by western blot analysis **(C)** and calculated **(D)**. The data were represented as means ± SEM (*n* = 8). ^∗^*p* < 0.05 vs. Sham. ^#^*p* < 0.05 vs. Vehicle.

Mitochondrial dynamics are also involved in mitochondrial dysfunction under oxidative stress. As shown in Figure 8A, we detected the expression of mitochondrial dynamic proteins in spinal cord tissues after SCI/R at different time points. The results showed that SCI/R decreased the expression of Opa-1 and Mfn-1 at 12 and 24 h but increased the expression of Fis-1 from 3 to 24 h, with no effect on Drp-1 expression (Figure 8B). Furthermore, we detected the expression of these proteins after NS309 treatment (Figure 8C). We found that the decreased expression of Opa-1 and Mfn-1, as well as increased expression of Fis-1 after SCI/R, were all partially prevented by NS309 treatment (Figure 8D).

## Discussion

Due to the lack of data from randomized controlled clinical trials, contemporary concepts about SCI/R protection are mainly adapted from guidelines for brain ischemia, cardiovascular ischemia and spinal cord trauma (Nardone et al., 2016). This study provides evidence that NS309, a pharmacological activator for SK/K_Ca_ channel, protects against experimental SCI/R in rabbits. Our results showed that (a) SCI/R decreases SK/K_Ca_1 protein expression in spinal cord tissues; (b) NS309 attenuates neuronal loss and neurological dysfunction after SCI/R; (c) NS309 reduces oxidative stress and regulates the levels of inflammatory cytokines after SCI/R; (d) NS309 preserves mitochondrial function and promotes mitochondrial biogenesis; and (e) NS309 differently regulates mitochondrial dynamic proteins.

The SK/K_Ca_ channels are extensively expressed throughout the central nervous system, but the three subtypes are differentially expressed. SK/K_Ca_1 can be found in cortex, hippocampus, midbrain, cerebellum and spinal cord; SK/K_Ca_2 is localized in cortex, amygdala, medial habenula and the inferior olivary complex; SK/K_Ca_3 is expressed in olfactory bulbs, dentate gyrus thalamus and the locus coeruleus (Sailer et al., 2004). Thus, we detected the expression of SK/K_Ca_1 protein to investigate the effect of SCI/R on SK/K_Ca_ channels. Significant decrease in SK/K_Ca_1 expression was observed at 12 and 24 h after SCI/R, and this decrease was detected in both cytosolic and mitochondrial fractions. In neuronal cells, SK/K_Ca_ channels were originally thought to be predominantly presented in the plasma membrane where they regulates K^+^ efflux (Honrath et al., 2017). However, a previous study showed that SK/K_Ca_ channels were also located in the inner mitochondrial membrane of neuronal mitochondria (Dolga et al., 2013). In addition, Dolga et al. demonstrated that the SK/K_Ca_2 subtype expressed in mitochondrial-enriched fractions and co-localized with the mitochondrial markers in neuronal dopaminergic cells (Dolga et al., 2014). Our results here demonstrated that SK/K_Ca_1 was also localized in mitochondria in spinal cord, and was downregulated after SCI/R, indicating a potential role in mitochondrial function after SCI/R.

Activation of SK/K_Ca_ channels has been demonstrated to exert protective effects in many *in vitro* neuronal models, and is considered as an emerging therapeutic approach for the treatment of several neurological diseases (Anderson et al., 2006; Dolga et al., 2011, 2012, 2013, 2014; Dolga and Culmsee, 2012). NS309 is a selective and potent SK/K_Ca_ channel opener that activates SK/K_Ca_ channels via increasing their Ca^2+^ sensitivity (Strobaek et al., 2006). The EC_50_ of NS309 to SK/K_Ca_ channels is 30 nM, and it has been demonstrated to be safe and could activate SK/K_Ca_ channels in both *in vitro* and *in vivo* experimental models (Hipolito et al., 2015; Honrath et al., 2017). In this study, we found that NS309 treatment reduced spinal cord edema and spinal cord neuronal loss after SCI/R, which were accompanied by preserved neurological function. Our data extended the neuroprotective effects of SK/K_Ca_ channels activation into SCI/R. Importantly, we found that the protective effects of NS309 were still observed when it was administrated at 6 h after ischemia initiation. One of the serious problems of preclinical experiments is that many neuroprotective agents are administrated prior to injury initiation (Menon, 2009). It is of little clinical relevance due to the difficulty in obtaining informed consent. Our results showed that the therapeutic time window of NS309 was up to 6 h after ischemia initiation, which is useful for future clinical trials. This time window of administration makes it possible to treat SCI/R with NS309 associated neuroprotective agents. Considering the safety of NS309 treatment in previous experimental animal models, NS309 might be an ideal candidate for clinical drug research for SCI/R.

Free radicals-associated oxidative damage is one of the most important mechanisms underlying secondary injury after SCI/R, especially in the reperfusion phase (Chen X.P. et al., 2011). The overproduced ROS causes protein and lipid peroxidation, thereby leading to loss of membrane potential and corruption of membrane fluidity, which eventually results in the release of organelle content. In this study, increased levels of MDA and 8-iso-PGF2α, two oxidative products that correlate with the extent of ROS-mediated damage, was observed in both serum and spinal cord tissues after SCI/R, which were reduced by NS309. Neuronal fate under oxidative stress is dependent on the balance between ROS and the anti-oxidative defense mechanisms within the cell (Jia et al., 2012). SOD and CAT are two endogenous antioxidant enzymes, that can scavenge the superoxide radicals by changing the O_2_^-^ into less damaging species (Rodrigo et al., 2011). At 24 and 48 h after SCI/R, reduced activities of SOD and CAT was found to be preserved by NS309, indicating the anti-oxidative activity of NS309 in our model. Thus, the effects of NS309 on prevention of oxidative stress after SCI/R might be the direct result of the increased activity of antioxidant enzymes, which needs to be further determined in the future. Inflammation is a subsequent event of SCI/R, and inflammatory cell infiltration and release of inflammatory cytokines were observed in spinal cords (Zhu et al., 2013). The pro-inflammatory cytokines, such as TNF-α and IL-1β, and anti-inflammatory cytokines, including IL-10 and TGF-β1, play opposite roles in inflammation regulation (Spitzbarth et al., 2012). Here, NS309 treatment was shown to upregulate anti-inflammatory cytokines but downregulate pro-inflammatory cytokines in the spinal cord tissues. Thus, inhibition of inflammatory responses via differentially regulating inflammatory cytokines might also contribute to NS309-induced protection against SCI/R.

Considering that the SK/K_Ca_1 subtype was found in spinal cord mitochondria, and that NS309 alleviated oxidative stress after SCI/R, we speculated that activation of SK/K_Ca_ channels could regulate mitochondrial function in the spinal cord. Thus, we further measured mitochondrial complexes activity. It has been demonstrated that dysfunction of mitochondrial complexes occurred within 4–6 h after reperfusion and resulted in permanent mitochondrial damage (Sims and Muyderman, 2010). As expected, we found that the reduced activities of mitochondrial complexes after SCI/R, except for complex II, were all partially prevented by NS309. In addition, these effects were accompanied by the changes in mitochondrial biogenesis factors. Mitochondrial biogenesis is defined as the regulation of mitochondrial mass and turnover, a mechanism aimed to maintain diverse homeostatic demands under physiological and pathological conditions (Chen S.D. et al., 2011). It requires exquisite communication between the mitochondrial genome and the nuclear genome, and can be measured by the copy number and integrity of mtDNA, as well as the levels of many transcription factors, such as NRF-1, PGC-1, and TFAM (Kelly and Scarpulla, 2004). Previous studies have shown that these candidate mitochondrial biogenesis regulatory proteins correlate with mitochondrial respiratory capacity and could augment tolerance to ischemic injury via activating antioxidant system (McLeod et al., 2005). Thus, our results strongly indicate that NS309 might promote mitochondrial biogenesis, leading to inhibition of oxidative stress, and thereby induce protection against SCI/R.

Dynamic structural changes of the mitochondrial network, named as mitochondrial dynamics, are governed by the delicate balance between frequent fusion and fission events, and can adapt mitochondrial function to meet energy demand under stress (Otera et al., 2013). Impaired regulation of mitochondrial dynamic proteins contributes to ischemic injury via reducing energy production and promoting ROS generation (Calo et al., 2013). Multiple mitochondrial guanosine triphosphates hydrolases (GTPases) that regulate mitochondrial networking have been identified, among which DRP-1 and Fis-1 are related to mitochondrial fission, whereas mitochondrial fusion is mediated by Opa-1 and Mfn-1 (Karbowski et al., 2002; Hoppins et al., 2007). We found that SCI/R decreased the expression of Opa-1 and Mfn-1 but increased the levels of Fis-1 rather than Drp-1, indicating the induction of mitochondrial fragmentation in spinal cord after injury. In addition, the changes of these proteins after SCI/R were significantly prevented by NS309 treatment. Previous studies have shown that Opa-1 and Mfn-1 functionally interact to promote mitochondrial elongation, while Fis-1 was required in the recruitment of Drp-1 to mitochondria during mitochondrial fission (Cipolat et al., 2004; De Palma et al., 2010). However, no significant changes in Drp-1 expression after SCI/R and/or NS309 treatment was observed in this study. Thus, a Drp-1-independent mechanism after SCI/R might exist, which need to be further determined.

In conclusion, our present study demonstrated the neuroprotective effects of NS309, a pharmacological activator for SK/K_Ca_ channel, against SCI/R in rabbits, which may involve an integrated process of the suppression of oxidative stress and the preservation of mitochondrial function.

## Author Contributions

TC and Y-HW conceived and designed the experiments. JZ, L-KY, and W-LC performed the experiments. WL analyzed the data. TC and JZ wrote the paper.

## Conflict of Interest Statement

The authors declare that the research was conducted in the absence of any commercial or financial relationships that could be construed as a potential conflict of interest.

## References

[B1] Anderson N. J. Slough S. Watson W. P. (2006). In vivo characterisation of the small-conductance KCa (SK) channel activator 1-ethyl-2-benzimidazolinone (1-EBIO) as a potential anticonvulsant. *Eur. J. Pharmacol.* 546 48–53. 10.1016/j.ejphar.2006.07.007 16925994

[B2] Calo L. Dong Y. Kumar R. Przyklenk K. Sanderson T. H. (2013). Mitochondrial dynamics: an emerging paradigm in ischemia-reperfusion injury. *Curr. Pharm. Des.* 19 6848–6857. 10.2174/138161281939131127110701 23590157

[B3] Chen S. D. Yang D. I. Lin T. K. Shaw F. Z. Liou C. W. Chuang Y. C. (2011). Roles of oxidative stress, apoptosis, PGC-1alpha and mitochondrial biogenesis in cerebral ischemia. *Int. J. Mol. Sci.* 12 7199–7215. 10.3390/ijms12107199 22072942 PMC3211033

[B4] Chen X. P. Fu W. M. Gu W. (2011). Spinal cord stimulation for patients with inoperable chronic critical leg ischemia. *World J. Emerg. Med.* 2 262–266. 10.5847/wjem.j.1920-8642.2011.04.003 25215020 PMC4129719

[B5] Cipolat S. Martins de Brito O. Dal Zilio B. Scorrano L. (2004). OPA1 requires mitofusin 1 to promote mitochondrial fusion. *Proc. Natl. Acad. Sci. U.S.A.* 101 15927–15932. 10.1073/pnas.0407043101 15509649 PMC528769

[B6] De Palma C. Falcone S. Pisoni S. Cipolat S. Panzeri C. Pambianco S. (2010). Nitric oxide inhibition of Drp1-mediated mitochondrial fission is critical for myogenic differentiation. *Cell Death Differ.* 17 1684–1696. 10.1038/cdd.2010.48 20467441 PMC3050583

[B7] Dolga A. M. Culmsee C. (2012). Protective roles for potassium SK/K(Ca)2 channels in microglia and neurons. *Front. Pharmacol.* 3:196. 10.3389/fphar.2012.00196 23189056 PMC3505862

[B8] Dolga A. M. de Andrade A. Meissner L. Knaus H. G. Hollerhage M. Christophersen P. (2014). Subcellular expression and neuroprotective effects of SK channels in human dopaminergic neurons. *Cell Death Dis.* 5:e999. 10.1038/cddis.2013.530 24434522 PMC4040692

[B9] Dolga A. M. Letsche T. Gold M. Doti N. Bacher M. Chiamvimonvat N. (2012). Activation of KCNN3/SK3/K(Ca)2.3 channels attenuates enhanced calcium influx and inflammatory cytokine production in activated microglia. *Glia* 60 2050–2064. 10.1002/glia.22419 23002008 PMC3799773

[B10] Dolga A. M. Netter M. F. Perocchi F. Doti N. Meissner L. Tobaben S. (2013). Mitochondrial small conductance SK2 channels prevent glutamate-induced oxytosis and mitochondrial dysfunction. *J. Biol. Chem.* 288 10792–10804. 10.1074/jbc.M113.453522 23430260 PMC3624460

[B11] Dolga A. M. Terpolilli N. Kepura F. Nijholt I. M. Knaus H. G. D’Orsi B. (2011). KCa2 channels activation prevents [Ca2+]i deregulation and reduces neuronal death following glutamate toxicity and cerebral ischemia. *Cell Death Dis.* 2:e147. 10.1038/cddis.2011.30 21509037 PMC3122061

[B12] Faber E. S. Sah P. (2007). Functions of SK channels in central neurons. *Clin. Exp. Pharmacol. Physiol.* 34 1077–1083. 10.1111/j.1440-1681.2007.04725.x 17714097

[B13] Griepp R. B. Griepp E. B. (2007). Spinal cord perfusion and protection during descending thoracic and thoracoabdominal aortic surgery: the collateral network concept. *Ann. Thorac. Surg.* 83 S865–S869; discussion S890–S892. 10.1016/j.athoracsur.2006.10.092 17257943

[B14] Hipolito L. Fakira A. K. Cabanero D. Blandon R. Carlton S. M. Moron J. A. (2015). In vivo activation of the SK channel in the spinal cord reduces the NMDA receptor antagonist dose needed to produce antinociception in an inflammatory pain model. *Pain* 156 849–858. 10.1097/j.pain.0000000000000124 25734988 PMC4428572

[B15] Honrath B. Krabbendam I. E. Culmsee C. Dolga A. M. (2017). Small conductance Ca(2+)-activated K(+) channels in the plasma membrane, mitochondria and the ER: pharmacology and implications in neuronal diseases. *Neurochem. Int.* 109 13–23. 10.1016/j.neuint.2017.05.005 28511953

[B16] Hoppins S. Lackner L. Nunnari J. (2007). The machines that divide and fuse mitochondria. *Annu. Rev. Biochem.* 76 751–780. 10.1146/annurev.biochem.76.071905.090048 17362197

[B17] Jia Z. Zhu H. Li J. Wang X. Misra H. Li Y. (2012). Oxidative stress in spinal cord injury and antioxidant-based intervention. *Spinal Cord* 50 264–274. 10.1038/sc.2011.111 21987065

[B18] Karbowski M. Lee Y. J. Gaume B. Jeong S. Y. Frank S. Nechushtan A. (2002). Spatial and temporal association of Bax with mitochondrial fission sites, Drp1, and Mfn2 during apoptosis. *J. Cell Biol.* 159 931–938. 10.1083/jcb.200209124 12499352 PMC2173996

[B19] Kelly D. P. Scarpulla R. C. (2004). Transcriptional regulatory circuits controlling mitochondrial biogenesis and function. *Genes Dev.* 18 357–368. 10.1101/gad.1177604 15004004

[B20] Kuiper E. F. Nelemans A. Luiten P. Nijholt I. Dolga A. Eisel U. (2012). K(Ca)2 and k(ca)3 channels in learning and memory processes, and neurodegeneration. *Front. Pharmacol.* 3:107. 10.3389/fphar.2012.00107 22701424 PMC3372087

[B21] LeMaire S. A. Price M. D. Green S. Y. Zarda S. Coselli J. S. (2012). Results of open thoracoabdominal aortic aneurysm repair. *Ann. Cardiothorac. Surg.* 1 286–292. 10.3978/j.issn.2225-319X.2012.08.16 23977510 PMC3741780

[B22] McLeod C. J. Pagel I. Sack M. N. (2005). The mitochondrial biogenesis regulatory program in cardiac adaptation to ischemia–a putative target for therapeutic intervention. *Trends Cardiovasc. Med.* 15 118–123. 10.1016/j.tcm.2005.05.001 16039972

[B23] Menon D. K. (2009). Unique challenges in clinical trials in traumatic brain injury. *Crit. Care Med.* 37 S129–S135. 10.1097/CCM.0b013e3181921225 19104212

[B24] Nardone R. Pikija S. Mutzenbach J. S. Seidl M. Leis S. Trinka E. (2016). Current and emerging treatment options for spinal cord ischemia. *Drug Discov. Today* 21 1632–1641. 10.1016/j.drudis.2016.06.015 27326910

[B25] New P. W. McFarlane C. L. (2012). Retrospective case series of outcomes following spinal cord infarction. *Eur. J. Neurol.* 19 1207–1212. 10.1111/j.1468-1331.2012.03702.x 22435357

[B26] Otera H. Ishihara N. Mihara K. (2013). New insights into the function and regulation of mitochondrial fission. *Biochim. Biophys. Acta* 1833 1256–1268. 10.1016/j.bbamcr.2013.02.002 23434681

[B27] Panthee N. Ono M. (2015). Spinal cord injury following thoracic and thoracoabdominal aortic repairs. *Asian Cardiovasc. Thorac. Ann.* 23 235–246. 10.1177/0218492314548901 25178467

[B28] Rodrigo R. Miranda A. Vergara L. (2011). Modulation of endogenous antioxidant system by wine polyphenols in human disease. *Clin. Chim. Acta* 412 410–424. 10.1016/j.cca.2010.11.034 21130758

[B29] Sailer C. A. Kaufmann W. A. Marksteiner J. Knaus H. G. (2004). Comparative immunohistochemical distribution of three small-conductance Ca2+-activated potassium channel subunits, SK1, SK2, and SK3 in mouse brain. *Mol. Cell Neurosci.* 26 458–469. 10.1016/j.mcn.2004.03.002 15234350

[B30] Sims N. R. Muyderman H. (2010). Mitochondria, oxidative metabolism and cell death in stroke. *Biochim. Biophys. Acta* 1802 80–91. 10.1016/j.bbadis.2009.09.003 19751827

[B31] Spitzbarth I. Baumgartner W. Beineke A. (2012). The role of pro- and anti-inflammatory cytokines in the pathogenesis of spontaneous canine CNS diseases. *Vet. Immunol. Immunopathol.* 147 6–24. 10.1016/j.vetimm.2012.04.005 22542984

[B32] Stocker M. (2004). Ca(2+)-activated K+ channels: molecular determinants and function of the SK family. *Nat. Rev. Neurosci.* 5 758–770. 10.1038/nrn1516 15378036

[B33] Strobaek D. Hougaard C. Johansen T. H. Sorensen U. S. Nielsen E. O. Nielsen K. S. (2006). Inhibitory gating modulation of small conductance Ca2+-activated K+ channels by the synthetic compound (R)-N-(benzimidazol-2-yl)-1,2,3,4-tetrahydro-1-naphtylamine (NS8593) reduces afterhyperpolarizing current in hippocampal CA1 neurons. *Mol. Pharmacol.* 70 1771–1782. 10.1124/mol.106.027110 16926279

[B34] Ye R. Zhang X. Kong X. Han J. Yang Q. Zhang Y. (2011). Ginsenoside Rd attenuates mitochondrial dysfunction and sequential apoptosis after transient focal ischemia. *Neuroscience* 178 169–180. 10.1016/j.neuroscience.2011.01.007 21219973

[B35] Zhou L. Wang X. Xue W. Xie K. Huang Y. Chen H. (2013). Beneficial effects of hydrogen-rich saline against spinal cord ischemia-reperfusion injury in rabbits. *Brain Res.* 1517 150–160. 10.1016/j.brainres.2013.04.007 23603405

[B36] Zhu P. Li J. X. Fujino M. Zhuang J. Li X. K. (2013). Development and treatments of inflammatory cells and cytokines in spinal cord ischemia-reperfusion injury. *Med. Inflamm.* 2013:701970. 10.1155/2013/701970 23956505 PMC3728531

